# “A long bristling road full of adventure, full of discovery” gene therapy approaches in transfusion‐dependent thalassemia: The EHA‐EBMT consensus paper

**DOI:** 10.1002/hem3.70510

**Published:** 2026-10-17

**Authors:** Lucia De Franceschi, Emanuele Angelucci

**Affiliations:** ^1^ University of Verona and Azienda Ospedaliera Integrata Verona Italy; ^2^ IRCCS Ospedale Policlinico San Martino Genova Italy

“As you set out for Ithaka

hope your road is a long one,

full of adventure, full of discovery.”

*Ithaka* by Kostantinos Kavafis (Greek poet 1863−1933)

Thalassemia, sea (*thalassa*, Greek word) and blood (*emia*, Greek word), was originally identified in the countries surrounding the Mediterranean Sea. Thalassemia is a globally distributed monogenic red cell disorder, which global burden disease study (GBD) study estimates more than 1,300,000 cases worldwide.^1^, ^2^, ^3^ Progress in clinical management of transfusion dependent thalassemic (TDT) patients has improve the prevalence and the mortality, but TDT still negatively impacts patients' quality of life and survival.^1^, ^4^, ^5^ TDT is still largely managed with to the combination of chronic transfusion and iron chelation regimen. Recently, luspatercerpt, an activin ligan trap, and mitapivat, a pyruvate kinases activator, have increased the therapeutic options for TDT.^6^ Up to now the only curative treatments for TDT are allogeneic hemopoietic cell transplantation (HCT) and gene therapy, which was recently approved by both the European Medical Agency (EMA) and the food and drug administration (FDA). In Bone Marrow Transplantation journal, a joint action between the European Hematology Association (EHA) and the European Bone Marrow Transplantation (EBMT) generated a consensus paper on the identification of candidate with TDT for gene therapy.^7^ This is an update of the European Hematology Association (EHA), Scientific Working Group (SWG) on Red Cells and Iron, in collaboration with the Italian Society of Thalassemia and Hemoglobinopathies (SITE), which was focused on gene addition approach and published in 2021.^8^ The panel agreed that the large experience achieved and published in allogeneic HCT is the basis for developing the so far most accurate model to define priority criteria to access GT, since currently available GT requires a full myeloablative conditioning regimen and can be considered analogous to an autologous transplantation. Thus, the EHA‐EBMT panelists updated and revised the 2021 document discussing: (i) new available treatments; (ii) main data and progress on hematopoietic cell transplantation; (iii) results of clinical studies on both gene addition and gene editing in TDT patients, considering also the available follow‐up.^7^ The risk factors for early death and TDT‐related organ complications were also part of the literature revision and the panelists discussion. A special attention was devoted to the impact of organ damage related to the long‐term iron cytotoxity. The authors developed a novel flow‐chart for TDT patient selection for GT, introducing the central role of liver disease evaluation. This was based on the revision of the literature, the data in HCT and the recent acquisition on long term study in TDT population, showing that hepatic iron concentration provides a snapshot of both hepatic and total body iron overload; however, the critical factor is not the iron itself, but rather the iron‐related tissue damage that develops over years of oxidative stress resulting from poor control of iron toxicity.^9^ Therefore, assessing the risk associated with a therapeutic yet potentially toxic procedure requires determining the cumulative damage acquired, and hepatic fibrosis serves as a marker of years of accumulating oxidative stress.^7^, ^10^ This evaluates the history of liver disease as cumulative effects of iron accumulation/efficient iron chelation, hepatitis infections (e.g., HCV, HBV), alcohol consumption, and metabolic disorders (e.g., diabetes). The authors also identified TDT patients requiring invasive approach as liver biopsy to define liver disease before proceeding to GT and indicates the liver biopsy specimen to meet the criteria of accuracy and representativeness.^7^ The EHA‐EBMT document also discussed the impact of cardiomyopathy, endocrinopathy, nephropathy and thrombophilic status on TDT patient' selection. The panelists also identified noneligible patients and potential future candidate in respect to organ damage, iron overload, and age. Although the limited follow‐up available on TDT patients treated with GT, the authors designed a short‐medium and long‐term follow‐up, which aligns with EMA and FDA requirements and represents a cautious approach to an area of uncertainty. Indeed, using the words of the Greek poet K. Kavafis in Ithaka: “As you set out for Ithaka, hope your road is a long one, full of adventure, full of discovery” and as modern‐day Odysseus, we may be glimpsing the shores of Ithaka far off on the horizon, but a fierce wind could still drive us away. We still need to acquire more data on TDT patients' follow‐up in terms of number of patients treated and length of time of observation and to understand the impact of GT on patients' survival when compared to healthy population and the well‐established HCT. The EHA‐EBMT algorithm allowing for the prioritization of TDT patients who are most likely to benefit of GT therapy is much needed, not only because of the technical nature and high costs involved, but also because of the limited number of experienced centers capable of successfully performing the collection of a high number of CD34+ cells and properly manage both the drug product infusion and the potential complications occurring after treatment. Finally, the cost of GT therapy rises a problem of accessibility and equity for curative treatment particularly in low‐ and middle‐incoming countries (LMIC) with high incidence of TDT where even allo‐HSCT, although much less expensive, might be barely accessible due to economic, health infrastructures, and training issues. This contributes to increase the existing gap between high and LMIC in accessibility to innovative therapies going behind GT for hemoglobinopathies. In conclusion, the EHA‐EBMT working group on GT in TDT compete the series started with the algorithm on GT for patients with SCD.^11^ This is an excellent example of fruitful collaboration between experts from different scientific societies on hot issues with still limited data. In addition, the EHA‐EBMT consensus paper is also important for the scientific community, for less expert centers, and for expert centers to improve the follow‐up strategies (e.g., introduction of LSM determination in TDT follow‐up). This document will be regularly updated offering to the scientific community a useful and handy tool for making decision process in real life.

## AUTHOR CONTRIBUTIONS

**Lucia De Franceschi**: Conceptualization; writing—original draft; writing—review and editing. **Emanuele Angelucci**: Conceptualization; writing—original draft; writing—review and editing.

## CONFLICT OF INTEREST STATEMENT

Lucia De Franceschi: Research grants from Agios and Bristol; consultant for Roche. Emanuele Angelucci: Data Monitoring Committee Chair for Vertex and BMS; DMC member for Vifor; consultant for Menarini‐Stemline and Sanofi; participation in advisory board for Regeneron; received travel support from AbbVie, Sanofi and GILEAD.

## FUNDING

This research received no funding.

## Data Availability

Data sharing not applicable to this article as no datasets were generated or analyzed during the current study.
